# The Genomic Landscape of Actinic Keratosis

**DOI:** 10.1016/j.jid.2020.12.024

**Published:** 2021-07

**Authors:** Jason Thomson, Findlay Bewicke-Copley, Chinedu Anthony Anene, Abha Gulati, Ai Nagano, Karin Purdie, Gareth J. Inman, Charlotte M. Proby, Irene M. Leigh, Catherine A. Harwood, Jun Wang

**Affiliations:** 1 Centre for Cell Biology and Cutaneous Research, Blizard Institute, Barts and The London School of Medicine and Dentistry, Queen Mary University of London, London, United Kingdom; 2 Centre for Cancer Genomics and Computational Biology, Barts Cancer Institute, Queen Mary University of London, London, United Kingdom; 3 Department of Dermatology, The Royal London Hospital, Barts Health NHS Trust, London, United Kingdom; 4 Cancer Research UK Cambridge Institute, University of Cambridge, Cambridge, United Kingdom; 5 Cancer Research UK Beatson Institute, Glasgow, United Kingdom; 6 Institute of Cancer Sciences, University of Glasgow, Glasgow, United Kingdom; 7 Division of Molecular and Clinical Medicine, School of Medicine, University of Dundee, Dundee, United Kingdom

**Keywords:** AK, actinic keratosis, CNA, copy number aberration, cSCC, cutaneous squamous cell carcinoma, IC, immunocompetent, IS, immunosuppressed, LOH, loss of heterozygosity, SMG, significantly mutated gene, TMB, tumor mutational burden, WES, whole-exome sequencing

## Abstract

Actinic keratoses (AKs) are lesions of epidermal keratinocyte dysplasia and are precursors for invasive cutaneous squamous cell carcinoma (cSCC). Identifying the specific genomic alterations driving the progression from normal skin to skin with AK to skin with invasive cSCC is challenging because of the massive UVR-induced mutational burden characteristic at all stages of this progression. In this study, we report the largest AK whole-exome sequencing study to date and perform a mutational signature and candidate driver gene analysis on these lesions. We demonstrate in 37 AKs from both immunosuppressed and immunocompetent patients that there are significant similarities between AKs and cSCC in terms of mutational burden, copy number alterations, mutational signatures, and patterns of driver gene mutations. We identify 44 significantly mutated AK driver genes and confirm that these genes are similarly altered in cSCC. We identify azathioprine mutational signature in all AKs from patients exposed to the drug, providing further evidence for its role in keratinocyte carcinogenesis. cSCCs differ from AKs in having higher levels of intrasample heterogeneity. Alterations in signaling pathways also differ, with immune-related signaling and TGFβ signaling significantly more mutated in cSCC. Integrating our findings with independent gene expression datasets confirms that dysregulated TGFβ signaling may represent an important event in AK‒cSCC progression.

## Introduction

Actinic keratoses (AKs) are dysplastic epidermal keratinocyte lesions resulting from chronic UVR exposure. It is generally accepted that AKs are cutaneous squamous cell carcinoma (cSCC) premalignancies (Siegel et al., 2016): at least two-thirds of cSCC arise from AK, although fewer than 0.6% AK per year will progress to cSCC (Criscione et al., 2009).

Identifying genomic alterations driving AK‒cSCC progression is challenging because of the high level of background mutations in keratinocytes associated with UV exposure. cSCCs have an average tumor mutational burden (TMB) of 50 mutations per megabase DNA, making them among the most mutated of all human cancers (South et al., 2014). Epigenomic alterations and immunological factors (reviewed in Bottomley et al. [2019]) add further layers of complexity in characterizing this progression.

Several molecular genetic studies have examined AK and cSCC at the levels of gene expression, chromosomal instability, and mutations in known cancer genes. These studies generally show that AK and cSCC possess similar genetic alterations with some conflict on whether AKs are more or less genomically unstable (summarized in Supplementary Table S1). However, most AK‒cSCC genetic studies have used targeted techniques on fixed tissue, which have many limitations and biases. Only three previous studies have used whole-exome sequencing (WES) to study AK; these studies have included ≤10 samples and reported similar mutational spectra in AK and cSCC with frequent mutations in known cSCC tumor suppressor genes *TP53, NOTCH1, NOTCH2,* and *FAT1* (Albibas et al., 2018; Chitsazzadeh et al., 2016; Rodríguez-Paredes et al., 2018) (Supplementary Table S2).

In this study, we present the largest WES study of AK conducted to date and include AK from both immunocompetent (IC) and immunosuppressed (IS) patients. We demonstrate that AKs are strikingly similar to cSCC at the genomic level with similar patterns of driver genes and copy number alterations. We also demonstrate mutational signature 32 in all samples from patients exposed to azathioprine, providing further evidence for its role in keratinocyte carcinogenesis at the precursor stage. By integrating our mutational pathway analysis with independent gene expression datasets, we have identified that the dysregulation of TGFβ signaling may represent a critical gatekeeper pathway resulting in AK‒cSCC progression.

## Results

### Patients and samples

A total of 37 AKs from 37 patients (median age = 70 years, range = 48–86 years) were included (Supplementary Table S3): 23 AKs from IS patients and 14 AKs from IC patients. WES data from cSCC samples previously analyzed for 16 of these patients were also available for comparison (Inman et al., 2018).

### Mutational burden and mutational signatures

**Figure 1:**
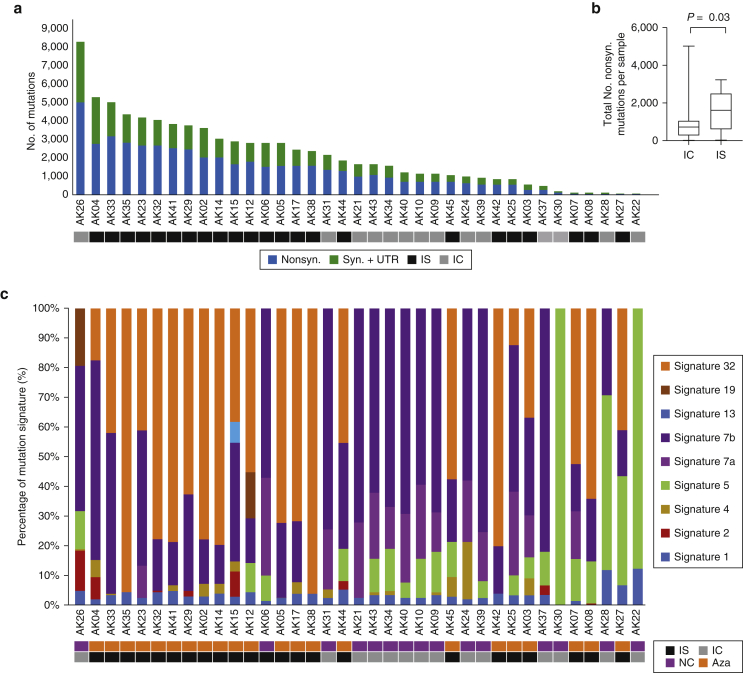
**The number of somatic mutations and mutation signatures across 37 AKs.** (**a**) No. of nonsyn., syn., and UTR mutations across the 37 samples. (**b**) Box and whisker plot comparing the total No. of nonsyn. mutations per AK from IS patients with those from IC patients, showing significantly more mutations in IS patients (Wilcoxon, *P =* 0.03). (**c**) Mutation signature compositions across the 37 AK samples. Signature 32 = azathioprine signature, and Signatures 7a/7b = UVR signatures. AK, actinic keratosis; Aza, confirmed azathioprine exposure; IC, immunocompetent; IS, immunosuppressed; NC, no confirmed azathioprine exposure; No., number; syn., synonymous; UTR, untranslated region.

WES targeted 334,378 exons from 20,965 genes and yielded a mean coverage of ×53 with 83% of targeted bases covered by ×20 and 94% of them covered by ×10. In total, 80,511 somatic mutations (range = 12–8,326 per AK) were identified. Of these, 49,853 were nonsynonymous (range = 9–4,993 per AK) with a median of 1,676 total and 1,071 nonsynonymous mutations per AK (Figure 1a and Supplementary Tables S4 and S5). This corresponds to a mean TMB of 43.5 mutations per megabase DNA, similar to the TMB of 50 mutations per megabase DNA we previously reported in cSCC (Inman et al., 2018).

AKs from IS patients were significantly more mutated than those from IC patients when comparing total nonsynonymous mutations (median = 1,609 vs. 729 mutations, respectively; Wilcoxon test, *P* = 0.03; Figure 1b). The results remained significant when controlled for per sample read depth (Supplementary Table S5). AKs from IS patients had a significantly higher median TMB than those from IC patients (55.9 and 22.3 mutations per megabase DNA, respectively; Wilcoxon test, *P =* 0.03). There was no significant difference in nonsynonymous mutation rates from AK compared with those from cSCC (median = 1,070 vs. 912; Wilcoxon test, *P =* 0.32).

Nine single-base substitution mutational signatures (Alexandrov et al., 2020, 2013) were identified (Figure 1c). A total of 33 AKs (89%) displayed characteristic UVR signatures (7a/b, 13–97%), mainly C > T transitions at dipyrimidine sites. A total of 20 AKs (54%) harbored variable levels (5–100%) of signature-5 mutations, and 35 AKs (95%) had low levels of signature-1 mutations (1–13%). The etiologies of signatures 5 and 1 are unknown, but both are commonly found at low levels in most cancers and are clock-like because the number of mutations correlates with age.

Signature 32 was detected in 22 AKs (59%), exclusively in patients exposed to azathioprine (Figure 1c) (Fisher’s exact test, *P* < 0.00001), reproducing our findings in cSCC. In contrast to cSCC, the prevalence was not significantly associated with the duration of azathioprine exposure (*r* = 0.21, *P* = 0.33, Supplementary Table S6).

### Somatic copy number aberrations

**Figure 2:**
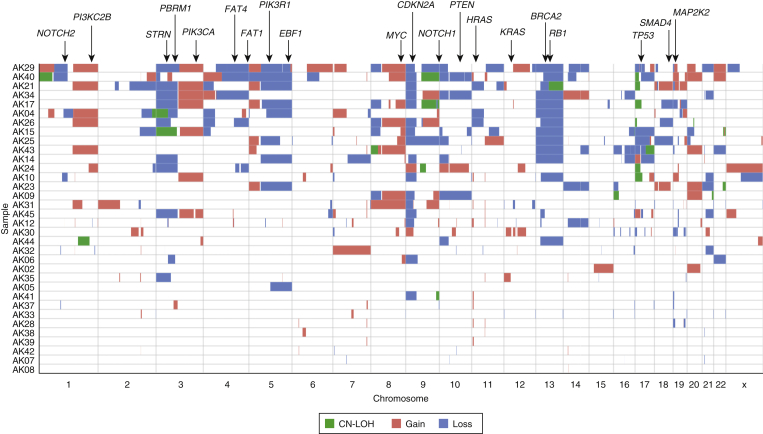
**Somatic CNA and LOH events in 37 AKs.** OncoPrint of copy-gain, copy-loss, and CN LOH segments with selected known cancer driver genes annotated earlier. AK, actinic keratosis; CN, copy-neutral; CNA, copy number aberration; LOH, loss of heterozygosity.

Copy number aberrations (CNAs) and loss of heterozygosity (LOH) events were also examined (Figure 2 and Supplementary Table S7). Across our AK cohort, a median of 9% (range = 0–62%) of each AK genome was affected by CNAs (Supplementary Table S8), similar to cSCC (Wilcoxon test, *P* = 0.68) and independent of immune status (Wilcoxon test*, P =* 0.26). There was no significant correlation between mutational burden and the proportion of the genome affected by CNAs (*r =* 0.25, *P =* 0.13).

Loss of chromosome region 9p (43%), 13q (32%), 3p (24%), and 5q (24%) were the most frequent copy number losses; gain of 3q (19%), 8q (19%), 5p (16%), chromosome 20 (16%), and 1q (14%) were the most frequent gains. The most common AK CNAs correlated significantly with those of cSCC (*r* = 0.68, *P* = 0.003, Supplementary Table S9).

**Supplementary Figure S1:**
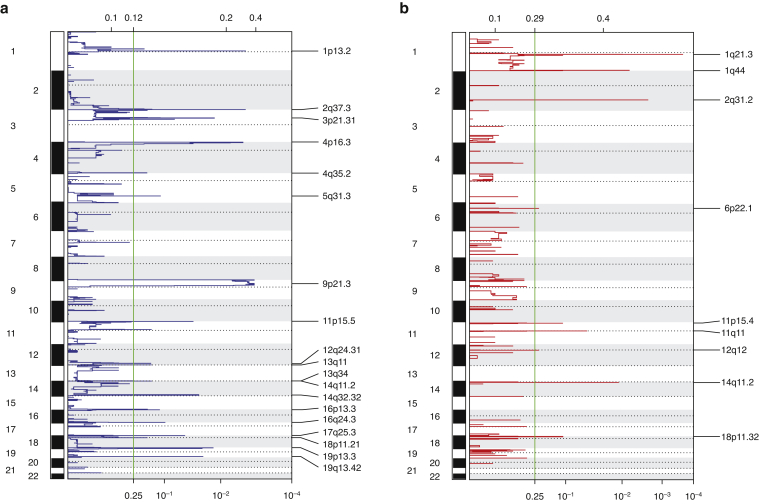
**Significantly (a) deleted and (b) gained regions across the 33 AK samples based on GISTIC2.0.** Four samples were excluded from the analysis because they have too few CNA segments. AK, actinic keratosis; CNA, copy number aberration.

GISTIC (Mermel et al., 2011) analysis identified 19 significantly deleted regions (*q* < 0.25) encompassing 859 genes, including 27 known cancer genes (Catalogue Of Somatic Mutations In Cancer database [Tate et al., 2019]). 9p21.3 was the most significantly deleted segment in AK and harbors 10 cancer genes, including *CDKN2A, JAK2,* and *MLLT3* (Supplementary Figure S1a and Supplementary Table S10). Nine regions were significantly gained (*q* < 0.25) encompassing 59 genes, none known to be cancer related (Supplementary Figure S1b and Supplementary Table S11). Analysis in IC and IS subgroups did not resolve any major differences because of the limited power within each group (Supplementary Table S12).

### Significantly mutated genes in AK

**Figure 3:**
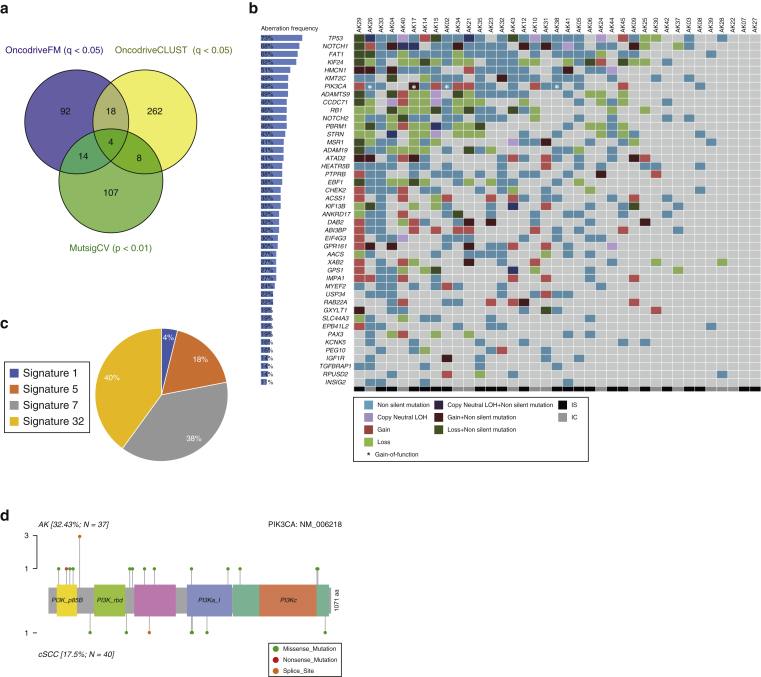
**A total of 44 SMGs identified in the 37 AKs.** (**a**) Venn diagram showing the overlap of numbers of SMGs as assessed by MutsigCV, OncodriveFM, and OncodriveCLUST. (**b**) Mutation OncoPrint of the 44 SMGs identified by at least two of the three methods, with CNA integrated and overall alteration frequency indicated with percentage bars. (**c**) Pie chart of the mutational signature contribution to 44 SMG mutations. (**d**) Lollipop plot of PIK3CA showing the distribution of mutation in AK and cSCC cohorts. aa, amino acid; AK, actinic keratosis; CNA, copy number aberration; cSCC, cutaneous squamous cell carcinoma; IC, immunocompetent; IS, immunosuppressed; LOH, loss of heterozygosity; PI3K, phosphoinositide 3-kinase; SMG, significantly mutated gene.

We used three methods to identify significantly mutated genes (SMGs): MutsigCV (*P* < 0.01) (Lawrence et al., 2013), OncodriveFM (*q* < 0.05) (Gonzalez-Perez et al., 2012), and OncodriveCLUST (*q* < 0.05) (Tamborero et al., 2013). A total of 44 SMGs were confirmed by at least two methods (Figure 3a and b and Supplementary Table S13) and included the tumor suppressor genes consistently reported as mutated in cSCC *(TP53, NOTCH1, NOTCH2, FAT1,* and *KMT2C)*. *HMCN1* was also altered in 50% of AKs and cSCC, consistent with previous smaller studies (Albibas et al., 2018; Durinck et al., 2011; Pickering et al., 2014). Additional SMGs included *CHEK2*, *PBRM1*, *PTPRB*, *EBF1*, *STRN*, and *PAX3*. All of them are causally implicated in other cancers. *PIK3CA* was the only oncogene significantly mutated using all the three methods: nonsynonymous mutations were present in 12 AKs (32%), with several of them targeting the N-terminal portion, including one nonsense and three missense mutations in the PI3K_p85B domain and a hotspot splicing mutation (n = 3) just downstream of this domain (Figure 3d). Five AK mutations (including the splice-site hotspot) are known gain-of-function oncogenic mutations (Supplementary Table S14) (Chakravarty et al., 2017).

Other SMGs (*IGF1R*, *ATAD2, ABI3BP, MSR1, ADAMTS9, ADAM19, KIF13B, DAB2, EIF4G3, GPR161, USP34, KCNK5, ACSS1, TGFBRAP1, RAB22A, XAB2, PEG10, IMPA1)* have all been associated with other cancers: mutations in all of them except in *RAB22A* are reported in cSCC in the Catalogue Of Somatic Mutations In Cancer database (https://cancer.sanger.ac.uk/cosmic [accessed 7 January 2020]) (Tate et al., 2019). The remaining 12 SMGs (*HEATR5B, KIF24, ANKRD17, AACS, MYEF2, GXYLT1, CCDC71, EPB41L2, GPS1, SLC44A3, INSIG2,* and *RPUSD2*) have no known role in other cancers but are found mutated in cSCC in the Catalogue Of Somatic Mutations In Cancer database. The frequency of mutations in SMGs was independent of the immune status, suggesting that AKs in both groups have common drivers (Supplementary Table S15). The majority of mutational signatures present in the 44 SMGs were signature 32 (40%) and signature 7 (38%) (Figure 3c), implicating azathioprine and UV exposure as key environmental carcinogens.

IS and IC subgroup analysis using our SMG pipeline identified 23 SMGs (detected by both OncodriveFM and MutsigCV, *P* < 0.05 cutoff was implemented because of smaller sample sizes) in the IS group and 10 SMGs in the IC group (Supplementary Tables S16 and S17). OncodriveCLUST was unsuccessful for this analysis. *TP53*, *NOTCH1*, and *NOTCH2* were the only SMGs common to IS, IC, and the combined cohort.

### Comparison between AK and cSCC SMGs

We integrated somatic mutations and CNAs to produce a mutation OncoPrint for the 44 AK SMGs (Figure 3b) and compared this with the OncoPrint for the 22 cSCC SMGs from our previous series. *TP53, NOTCH1,* and *NOTCH2* were the only shared SMGs, and 6 of the total 63 SMGs showed differed frequencies between AK and cSCC (Supplementary Table S18). *CCDC71L* (chi-square test, *P* = 0.004) and *STRN* (*P* = 0.014) were altered at higher frequency in AK, whereas *LCLAT1* (*P* = 0.032), *HERC6* (*P* = 0.029), *MAP3K9* (*P* = 0.043), and *TMEM51* (*P* = 0.048) were altered at a higher frequency in cSCC. When adjusted for multiple comparisons, none of the 63 SMGs were altered significantly between AK and cSCC.

**Supplementary Figure S2:**
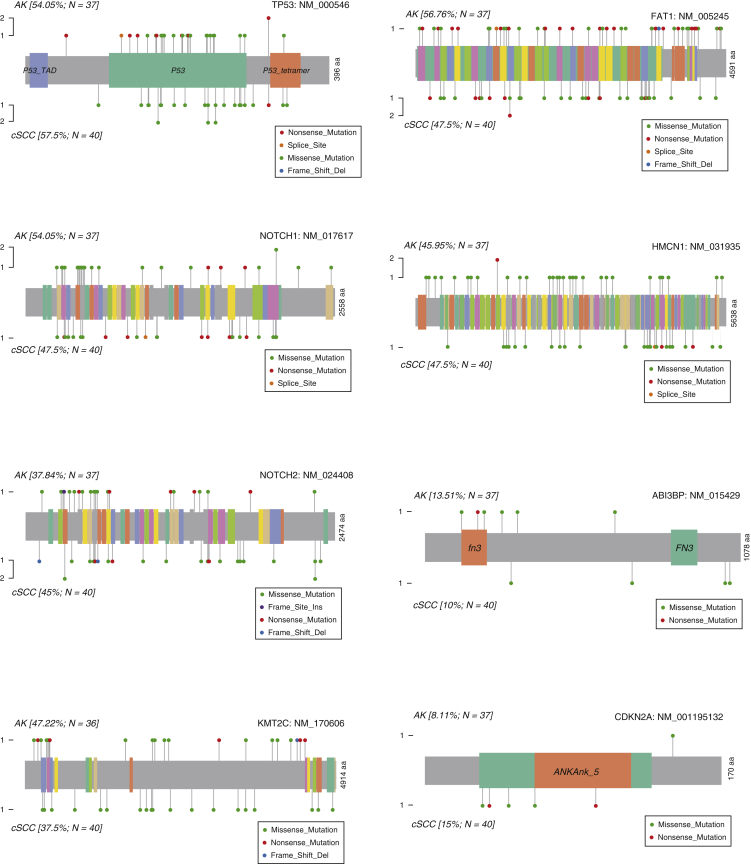
**Lollipop plots of key AK and cSCC driver genes showing the type and distribution patterns of nonsynonymous mutations between AK and cSCC.** aa, amino acid; AK, actinic keratosis; cSCC, cutaneous squamous cell carcinoma; Del, deletion; Ins, insertion.

We also compared AK SMGs with well-differentiated and moderately and/or poorly differentiated group-specific SMGs (Inman et al., 2018). There were no significant differences between SMGs in AK and those in well-differentiated cSCC, but 10 moderately and/or poorly differentiated cSCC SMGs were significantly more altered in cSCC than in AK (*PRB1, TMEM51, LRP1, POLH, ACVR2A, RPLP1, ZZEF1, VWF, ICAM1*, and *HECTD4*) (Supplementary Table S18). The mutation distribution along the protein domains was similar for AK and cSCC SMGs (Supplementary Figure S2), with the exception of the *PIK3CA* hotspot in AK, as described earlier.

Genes previously implicated in cSCC, such as *CDKN2A* and *HRAS*, were found altered at similar rates between our AK and cSCC cohorts (54.1% and 45% altered for *CDKN2A*, respectively, and 16.2% and 22.5% altered for *HRAS*, respectively; Supplementary Table S18).

**Supplementary Figure S3:**
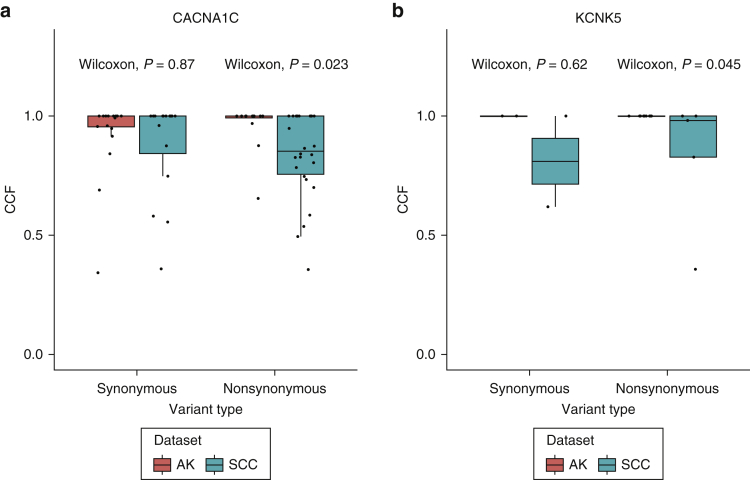
**Boxplots of CCFs of synonymous and nonsynonymous mutations of SMGs between AK and cSCC, highlighting that two genes had more clonal nonsynonymous mutations in AK than in (a) CACNA1C and (b) KCNK5, using synonymous mutations as the negative control.** AK, actinic keratosis; CCF, cancer cell fraction; SMG, significantly mutated gene; SCC, squamous cell carcinoma.

We next compared the cancer cell fractions of nonsynonymous mutations between AK and cSCC across the 63 SMGs to assess their clonality and to identify whether mutations in any genes are more clonally dominant in AK or cSCC using synonymous and untranslated region mutations as a negative control. Two genes (*CACNA1C* and *KCNK5*) showed significant evidence that nonsynonymous mutations in them were more clonally dominant in AK than in cSCC, suggesting that these genes may be under direct selection in the AK lineage (Supplementary Table S19 and Supplementary Figure S3).

### Inference of clonal evolution and order of genetic changes

**Figure 4:**
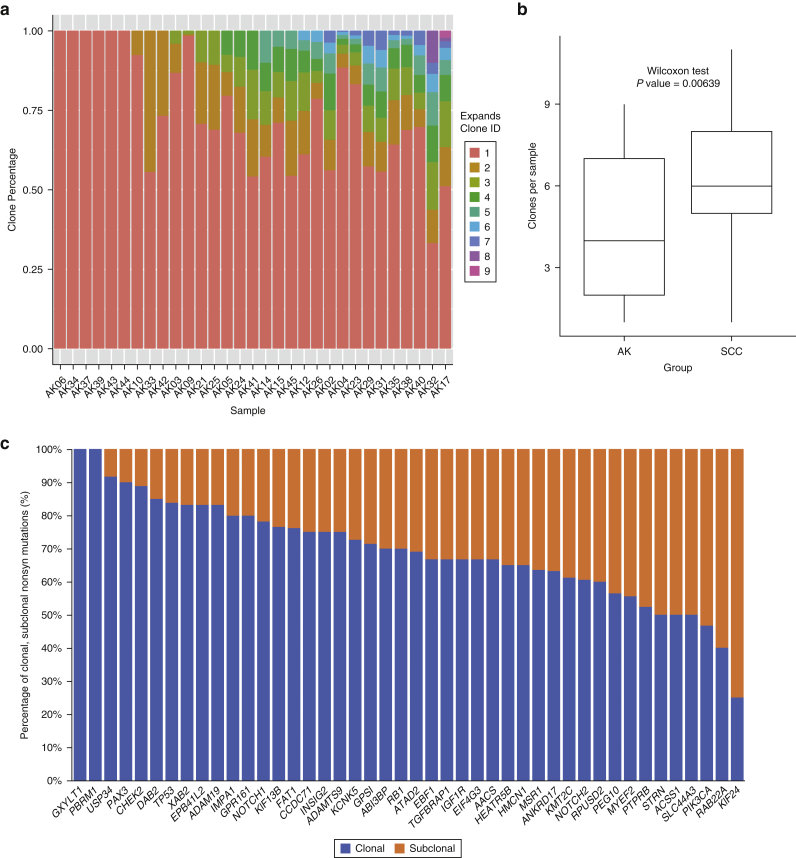
**Clonality analysis in AK.** (**a**) EXPANDS clonality analysis showing clones and their percentages per sample. (**b**) Box and whisker plot comparing clone sizes between AK and cSCC. *P* = 0.00639. (**c**) Clonality analysis of 44 SMGs identifying clonal and subclonal nonsyn mutations using EXPANDS. AK, actinic keratoses; cSCC, cutaneous squamous cell carcinoma; EXPANDS, Expanding Ploidy and Allele-frequency on Nested Subpopulations; ID, identity; SCC, squamous cell carcinoma; SMG, significantly mutated gene; syn, synonymous.

To assess the levels of intrasample heterogeneity, we performed clonality analyses using Expanding Ploidy and Allele-frequency on Nested Subpopulations (Andor et al., 2014), which estimates the number of clones and their proportions within each sample. A total of 31 AKs (84%) had sufficient numbers of somatic mutations (≥200) for analysis. Variation in intrasample heterogeneity was identified, with a median of four (range = 1–9) clones per AK (Figure 4a) compared with a median of six in cSCC (Wilcoxon test, *P* < 0.01, Figure 4b).

For each of the 44 SMGs, we assessed the order of mutation acquisition inferred from the aggregate frequencies at which they were found to be clonal or subclonal. More than 70% of mutations in 22 SMGs (*TP53, NOTCH1, FAT1, ADAMTS9, CCDC71, RB1, PBRM1, ADAM19, CHEK2, KIF13B, DAB2, ABI3BP, GPR161, XAB2, GPS1, IMPA1, USP34, GXYLT1, EPB41L2, PAX3, KCNK5,* and *INSIG2)* were clonal, indicating that alterations in these genes are more likely to be early events in AK development. Compared with the 22 SMGs mentioned earlier, a relatively larger proportion of subclonal nonsynonymous mutations, implying later development, were found in the remaining 22 SMGs, although more clonal than subclonal mutations were still observed for most of them, except for *PIK3CA*, *RAB22A,* and *KIF24* (Figure 4c). For the three SMGs shared by both AK and cSCC, patterns of clonality were similar, with a larger proportion of clonal mutations observed in *TP53* and *NOTCH1* than in *NOTCH2*.

**Supplementary Figure S4:**
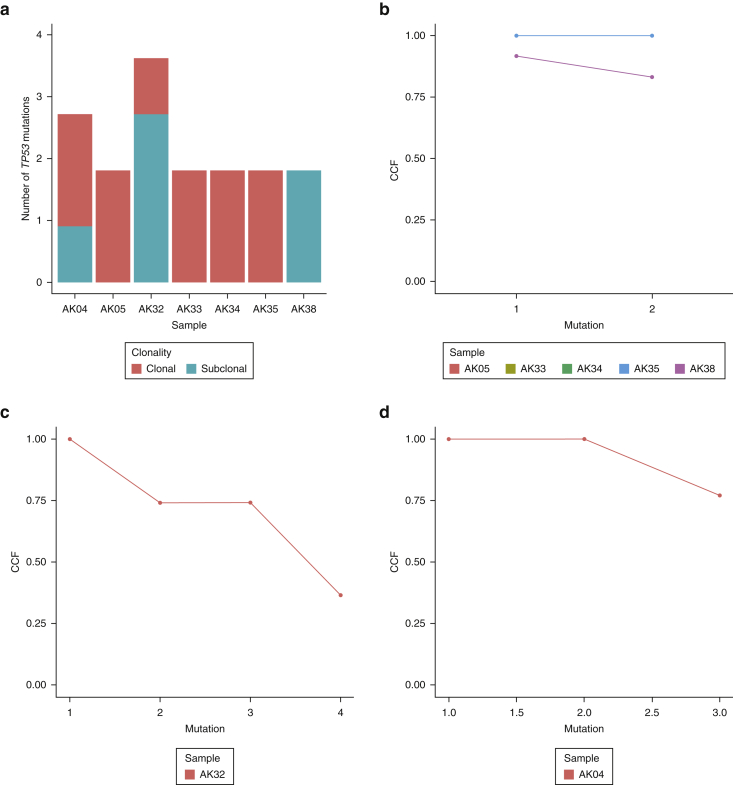
**TP53 clonality analysis.** (**a**) Bar chart of the number of clonal and subclonal *TP53* mutations in selected AK samples with >1 nonsynonymous TP53 mutation. (**b–d**) Line graphs of CCF of *TP53* nonsynonymous mutations for (**b**) AK05, AK33, AK34, AK38, (**c**) AK32, and (**d**) AK04. AK, actinic keratosis; CCF, cancer cell fraction.

Seven AKs had more than one nonsynonymous mutation in *TP53*, and we estimated the timing of multiple mutations in these lesions. In four samples (AK05, AK33, AK34, and AK35), all *TP53* mutations appeared to be clonal and thus likely early events on the basis of clonality analysis. Two samples (AK04 and AK32) showed a mixture of early and late (i.e., subclonal) events, and one sample (AK38) exhibited both *TP53* mutations as late events (Supplementary Figure S4a–d). These data suggest that although *TP53* mutations are commonly observed as early events, additional mutations can occur later on in the development of AK.

### Comparison of intrapatient AK with cSCC

**Supplementary Figure S5:**
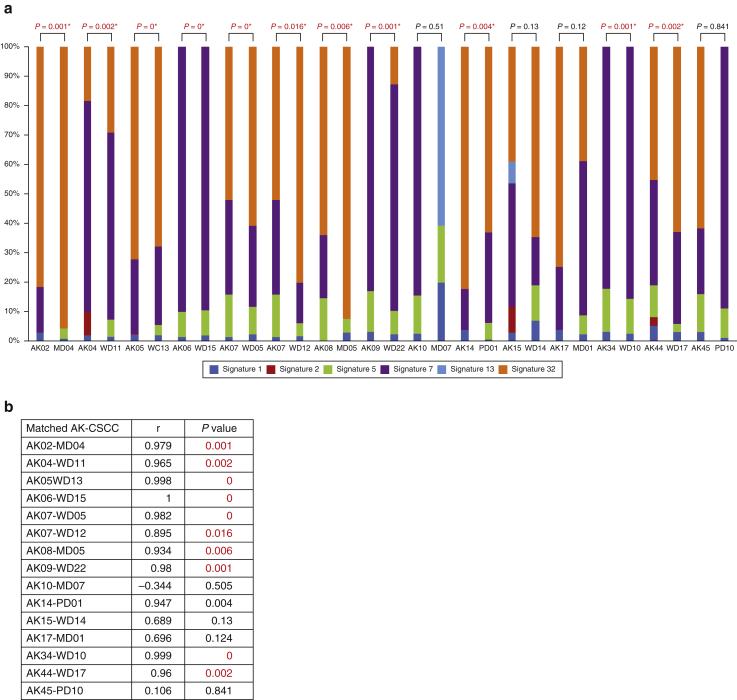
**Correlation of mutation signatures between matched AK and cSCC pairs.** (**a**) Mutation signature compositions in matched AK and cSCC pairs. Two pairs (AK03‒MD03 and AK29‒WD08) were not included because the cSCC did not have mutational signature data. *P*-values from Pearson correlation statistic. Signature 32 = azathioprine signature, and signature 7 = UVR signature. (**b**) Table of Pearson correlation analysis. AK, actinic keratosis; cSCC, cutaneous squamous cell carcinoma; MD, moderately differentiated; WD, well-differentiated.

A total of 16 AKs (43%) also had cSCC WES from the same individual (mostly from anatomically distinct sun-exposed fields) (Supplementary Table S20). There was a significant positive correlation in the mutational signature profiles of 71.4% (10 of 14) of the AK‒cSCC pairs for which data were available (Supplementary Figure S5a and b). Seven AK‒cSCC pairs had overlapping CNA segments involving a median of 20% of total CNA segments, most commonly present on chromosomes 3, 9, and 17. The direction of CNAs was largely concordant, although not statistically significant (chi-square test, *P =* 0.06) (Supplementary Table S21).

### Mutational pathway analysis

**Figure 5:**
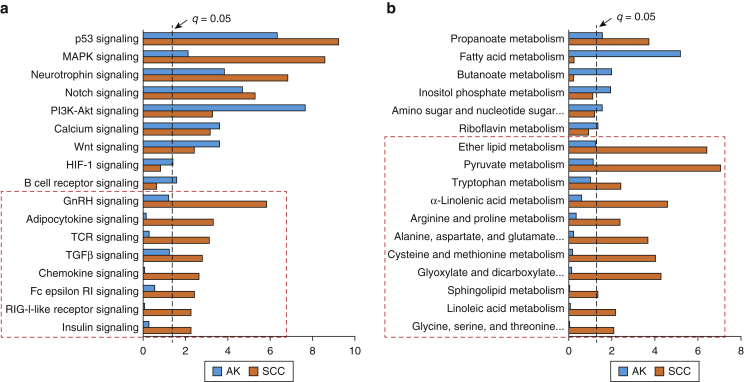
**Comparison of significantly mutated KEGG pathways between AK and cSCC.** (**a**) Significantly mutated signaling pathways derived from OncodriveFM. (**b**) Significantly mutated metabolic pathways. Pathways and biological processes in the bar charts were sorted with terms significant (cutoff *q* = 0.05) in both groups at the top and terms significant in cSCC only at the bottom (indicated with red dashed box). AK, actinic keratosis; Akt, protein kinase B; cSCC, cutaneous squamous cell carcinoma; KEGG, Kyoto Encyclopedia of Genes and Genomes; PI3K, phosphoinositide 3-kinase; SCC, squamous cell carcinoma.

We compared significantly mutated pathways between AK and cSCC (Figure 5a and b). Many immune system signaling pathways were significantly more mutated in cSCC than in AK (TCR, Fc epsilon-RI, RIG-I-like receptor, and chemokine signaling). TGFβ, adipocytokine, GnRH, and insulin signaling were also significantly more mutated in cSCC (Figure 5a). Several metabolism pathways were differentially mutated: ether lipid, pyruvate, or α-linolenic among others were significantly more mutated in cSCC (Figure 5b).

### Integration of genomic drivers, CNAs, and gene expression profiles

**Figure 6:**
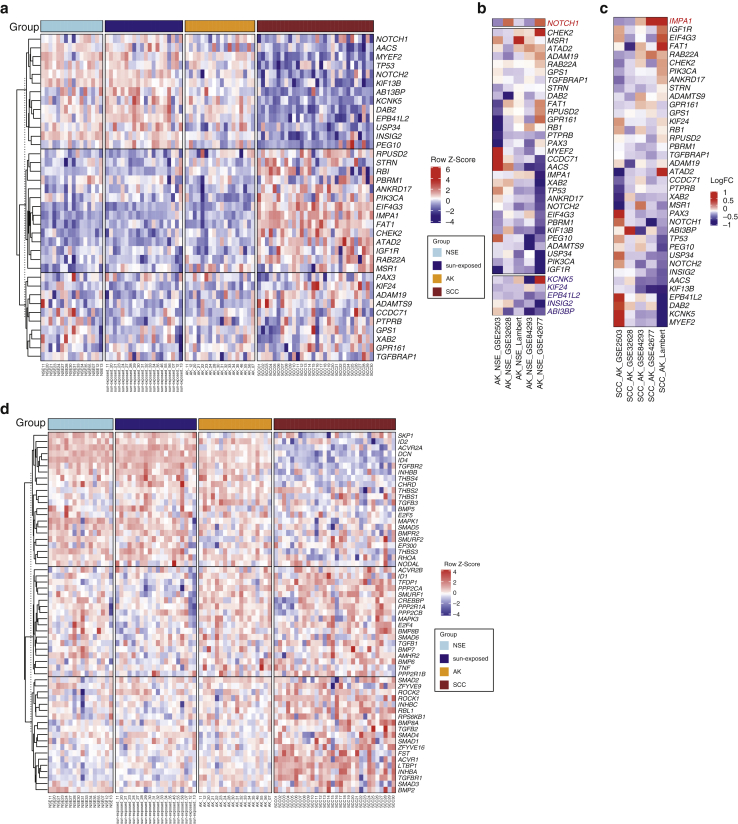
**Expression profiles of SMGs in the five independent gene expression datasets.** (**a**) Expression heatmap of 37 SMGs across NSE skin, sun-exposed skin, AK, and cSCC samples from the Lambert dataset. LogFC of pairwise comparisons of (**b**) AK versus NSE and (**c**) SCC versus AK across the five datasets (Lambert, GSE42677, GSE84293, GSE2503, and GSE32628). Normal skin: NSE skin, sun-exposed skin, AK, and SCC‒cSCC. Significant genes with the same direction in 2 of 5 datasets were highlighted in blue (downregulated) or in red (upregulated). Within **b** and **c**, the blue color indicates the downregulation in AK or SCC compared with the downregulation in NSE or AK, respectively, and the red color indicates the upregulation in AK or SCC relative to that in NSE or AK, respectively. (**d**) Expression heatmap of TGFβ signaling genes that were expressed across NSE skin, sun-exposed skin, AK, and cSCC samples from the Lambert dataset. AK, actinic keratosis; cSCC, cutaneous squamous cell carcinoma; LogFC, Log_2_ fold change; NSE, non–sun-exposed; SCC, squamous cell carcinoma; SMG, significantly mutated gene.

We integrated the existing gene expression profiles of normal skin, AK, and cSCC to investigate the potential tumor-suppressing or -promoting roles of AK SMGs through the pattern of their expression in disease progression. We used five independent datasets (described in the Materials and Methods section). The SMG hierarchies across the datasets were broadly concordant with two main clusters observed: one consisting of SMGs upregulated in normal skin and progressively downregulated in AK and cSCC, and the other cluster demonstrating the reverse pattern (Figure 6a).

**Supplementary Figure S6:**
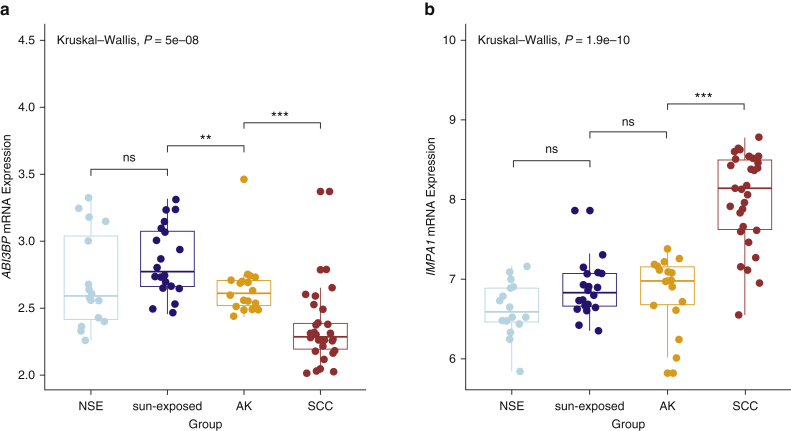
**Box and whisker plot comparing (a) *ABI3BP* with (b) *IMPA1* mRNA expression across NSE skin, sun-exposed skin, AK, and cSCC, depicting the statistics for between-group comparisons.** ns and significant at ∗∗*P* < 0.01 and ∗∗∗*P*< 0.001. AK, actinic keratosis; cSCC, cutaneous squamous cell carcinoma; ns, not significant; NSE, non–sun-exposed; SCC, squamous cell carcinoma.

In AK versus normal skin, five SMGs were significantly downregulated in at least two datasets (*KIF24, KCNK5, EPB41L, INSIG2,* and *ABI3BP)*, suggesting a tumor suppressor role (Figure 6 and Supplementary Figure S6a). *NOTCH1* was the only significantly upregulated SMG (Figure 6b). In cSCC versus AK, *IMPA1* was the sole SMG to be significantly differentially expressed in at least two datasets, being upregulated in cSCC, suggesting a tumor promoter role (Figure 6c and Supplementary Figure S6b).

Because the TGFβ signaling pathway was significantly more mutated in cSCC than in AK, we examined the expression patterns of TGFβ pathway genes in normal skin, in skin with AK, and in skin with cSCC. The datasets show two clusters of progression-dependent dysregulation of TGFβ signaling (Figure 6d). One cluster showed an upregulation of genes in the normal skin that become increasingly more downregulated, progressing from AK to cSCC, and the second cluster had the reverse pattern (Figure 6d).

**Supplementary Figure S7:**
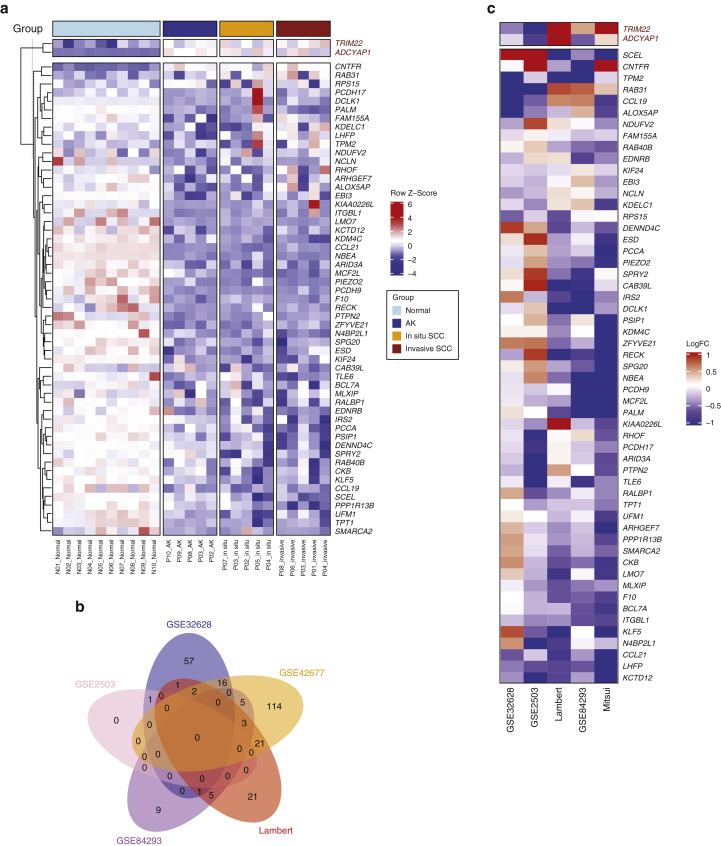
**Expression profiles of genes significantly gained (red) or deleted (black) in AK.** (**a**) Expression heatmap of significantly gained, upregulated genes (n = 2, red) and deleted, downregulated genes (n = 52, black) in the AK versus those in the normal skin that were also expressed across normal skin, AK, and in situ and invasive cSCC samples from the GSE42677. (**b**) Venn diagram of overlap across the five gene expression datasets of significantly deleted and downregulated genes in AK versus those in the normal skin. (**c**) LogFC of significant genes with the same direction in 2 of 5 datasets in pairwise comparisons of AK with normal skin across the five datasets (Lambert, GSE42677, GSE84293, GSE2503, and GSE32628). The blue color indicates the downregulation in AK compared with that in the normal skin, and the red color indicates the upregulation in AK relative to that in the normal skin. AK, actinic keratosis; cSCC, cutaneous squamous cell carcinoma; LogFC, Log_2_ fold change.

We assessed the gene expression of significantly deleted and gained regions identified by our GISTIC analysis, which revealed 55 genes in the deleted regions that were downregulated (in at least two datasets) and two genes in the gained regions that were upregulated (in at least two datasets) in AK compared with those in the normal control (Supplementary Figure S7a–c).

## Discussion

This study, the largest AK genomic dataset to date, demonstrates that AKs and cSCC are strikingly similar at the genomic level in terms of TMB, patterns of driver genes, and CNAs. The AK TMB of 43.5 mutations per megabase DNA is higher than previously reported (Albibas et al., 2018; Chitsazzadeh et al., 2016), which is likely a consequence of having a majority of the AKs from IS individuals because we have demonstrated immunosuppression results in significantly higher mutation rates. The TMB from the AKs of IC patients (30.4) is similar to the 34.5 reported previously (Albibas et al., 2018).

We detected the expected UV signature (7a/b) in most AKs. Furthermore, signature 32 was present in all AKs from patients exposed to azathioprine, further implicating this drug in the early stages of cSCC development (Inman et al., 2018). The significant positive correlation in the matched AK‒cSCC mutation profiles also provides further evidence that the same underlying mutational processes are at play in both AK and cSCC even when they are from different anatomical sites.

We identified 44 AK SMGs, including many classical tumor suppressor genes (*TP53, NOTCH1, NOTCH2,* and *FAT1*), that are consistently mutated in cSCC, but importantly, these genes are also mutated at low levels and under strong positive selection in physiologically normal sun-exposed skin (Martincorena et al., 2015). However, *CDKN2A* is not mutated in the normal skin, and we previously postulated that it may have a gatekeeper role in cSCC (Inman et al., 2018). *CDKN2A* was not identified as an SMG, but the loss of 9p21.3—a *CDKN2A* gene locus—was identified as the most significantly deleted CNA, and there was no significant difference in the frequency of deletion in AK compared with that in cSCC (54% vs. 45%, respectively, chi-square test, *P =* 0.43, Supplementary Table S18), suggesting that this deletion is an early event and may play an important role in AK pathogenesis. The oncogene *PIK3CA* was significantly altered in almost half of all AKs and cSCC, a higher frequency than seen in previous cSCC studies (Hafner et al., 2010; Janus et al., 2017; Pickering et al., 2014). We also identified a hotspot activating splice-site mutation in three AKs. Activating mutations in *PIK3CA* result in the activation of the phosphoinositide 3-kinases/protein kinase B/mTOR pathway, which is commonly seen in other organs’ squamous cell carcinomas (Vivanco and Sawyers, 2002).

Although we identified 16.2% AKs with alterations (all CNAs, four losses, and two gains) in *HRAS*, no single-nucleotide variants were detected in our cohort. This aligns with other studies where oncogenic *RAS* mutations are very rare in UV-exposed skin and AKs and/or squamous cell carcinoma in situ (Chitsazzadeh et al., 2016; Martincorena et al., 2015; Zheng et al., 2020). In our cSCC cohort, 22.5% harbored alterations in *HRAS*, and this was identified as a cSCC SMG, but only three cSCCs were predicted to have activating mutations. This provides further evidence that human AKs and cSCCs are biologically distinct from murine 7,12-dimethylbenz[a]anthracene/12-O-trtradecanoylphorbol-13-acetate model lesions where activating *HRAS* mutations are common (Huang and Balmain, 2014). Nevertheless, activating *RAS* mutations still appear to play a functional role in cSCC development for a subset of cSCC tumors rather than in AK‒cSCC in situ development.

*HMCN1* was identified as an SMG, being altered in 19 AKs (51%). Although not a confirmed cancer gene, its role warrants further investigation because it is frequently mutated in cSCC (Durinck et al., 2011; Pickering et al., 2014) and AKs (Albibas et al., 2018). It is postulated to be involved in cancer cell invasion and metastasis (Timpl et al., 2003) through its function as an extracellular matrix protein.

We show that AKs have the same levels of genomic instability as cSCC with many shared CNAs between them, notably loss of 9p, chromosome 13, and 5q. Apart from an early study that suggested that AKs have more LOH than cSCC (Rehman et al., 1996), our findings are contrary to those of more recent studies that found that AKs have relatively few CNAs (García-Díez et al., 2019; Hameetman et al., 2013). However, most studies agree on the most common sites of chromosomal instability, with 9p loss—the region where *CDKN2A* is located—ubiquitous among AK studies (Ashton et al., 2003; García-Díez et al., 2019; Kanellou et al., 2008; Rehman et al., 1996).

Interrogating published expression datasets against our SMG list found *KIF24, KCNK5, EPB41L2,* and *ABI3BP* downregulated in AK compared with those in the normal skin, suggesting tumor suppressor roles. *ABI3BP* was significantly downregulated in cSCC compared with that in the normal skin in one previous study (Prasad et al., 2014) and similarly downregulated in the esophagus with squamous cell carcinoma compared with that in the normal esophagus (Jiang et al., 2018). It is a tumor suppressor in several cancers where it functions to promote cellular senescence (Wakoh et al., 2009) and has a role in cell‒substrate adhesion (Hodgkinson et al., 2013; Latini et al., 2008). *KCNK5,* a two-pore domain potassium channel, is in the top 1% of underexpressed genes in melanoma and top 5% of underexpressed genes in breast, colorectal, renal, and liver cancers, and there is increasing interest in the role of potassium channels in cancer (Williams et al., 2013). *NOTCH1* mRNA was significantly upregulated in AK compared with that in the normal skin (in two datasets), suggesting tumor promoter function, which contrasts with previous findings in cSCC where it is inactivated early in cSCC pathogenesis (South et al., 2014). We also observe early mutational inactivation of *NOTCH1* in AK, and subsequent loss of expression may facilitate progression from AK to cSCC. NOTCH proteins have opposing tumor suppressor and promoter roles in different cancer types and contexts (Ranganathan et al., 2011), and this requires further investigation in AK to cSCC progression.

We have previously shown that the dysregulation of TGFβ signaling through inactivation of its receptors in stem cells is an early driver event in cSCC pathogenesis and plays a likely tumor suppressor role (Cammareri et al., 2016). TGFβ signaling pathway genes were significantly more mutated in cSCC and were also dysregulated in expression datasets. From the GSE45216 dataset (the largest dataset), *TGFBR2* becomes progressively more underexpressed in the transition from normal skin through AK to cSCC, consistent with the previous studies that have demonstrated a key role for *TGFBR2* in TGFβ tumor‒suppressive function (Han et al., 2005). Taken together, these findings further support the hypothesis that TGFβ dysregulation is a critical step in AK‒cSCC transition.

Epigenomic alterations may also play a part in driving AK progression, and this is supported by the significant differences we have observed in AK and cSCC expression profiles. Recent work landscaping the methylomes of AK and cSCC has directly addressed this hypothesis. A complex nonlinear evolution of distinct DNA-methylation patterns during the progression of AK to cSCC and metastasis has been demonstrated by one group (Hervás-Marín et al., 2019), but others failed to show any differences in AK and cSCC methylomes (Rodríguez-Paredes et al., 2018). Epigenomic studies to date are limited, and further research is needed.

A limitation of our study is that although we clinically diagnosed and then histologically confirmed AK, we did not specifically grade AK and analyze them according to grades, that is, AK-I, -II, and -III. There is a possibility that each of these AK grades might have a different molecular profile. However, AKs are frequently heterogeneous histologically and may include combinations of AK I–III. Consequently, there is a possibility that small foci of AK III and/or cSCC in situ were included in these samples, and this may have affected the results. Laser capture microdissection of samples may have helped to minimize this risk.

In conclusion, our data demonstrate that AKs already possess the majority of genomic alterations present in cSCC. Significant molecular alterations, which we have uncovered and which may contribute to evolution from AK to cSCC, include alterations in key signaling pathways, particularly in TGFβ and immune system signaling; mutations in specific genes, including *ABI3BP* and *IMPA1*; and differences in intrasample heterogeneity. These will be the focus of future investigations of the molecular pathogenesis of AK progression.

## Materials and Methods

### Collection of patient samples and clinical data

The 4 mm punch biopsies of AK diagnosed clinically and confirmed histologically were obtained from participants after written informed consent was obtained. Processing of AK and venous blood for matched germline DNA and clinical data collection was done as previously described (Inman et al., 2018). The study was approved by the East of Scotland Research Ethics Service (reference: 08/S1401/69) and the East London and City Health Authority Local Ethics Committee and conducted according to the Declaration of Helsinki Principles.

### DNA extraction and genetic analysis

DNA extraction and genetic analysis were performed as previously described (Inman et al., 2018).

### WES data processing, somatic variant calling, annotation, and visualization

WES data were analyzed and annotated using our established pipeline (Inman et al., 2018). Maftools was used to summarize, visualize, and compare AK and cSCC Mutation Annotation Format files and to make lollipop plots of gene mutation distributions (Mayakonda et al., 2018). Mutation signatures across the exomes were identified on the basis of the nonnegative matrix factorization approach previously described (Alexandrov et al., 2020, 2013) using the Catalogue Of Somatic Mutations In Cancer mutational signatures.

### Identification of CNA and LOH using WES data

Analyses for CNA and LOH events from WES data were based on a combinational approach previously described (Okosun et al., 2016; Tawana et al., 2015). Genes targeted by copy-gain, copy-loss, and copy-neutral LOH in each sample were identified. Thresholds for CNA calls were fully described in our previous study (Inman et al., 2018).

### Tumor subpopulation identification and clonality analysis

Expanding Ploidy and Allele-frequency on Nested Subpopulations (Andor et al., 2014) was used to estimate clonal expansions and cellular frequency of each clonal and subclonal population as previously described (Inman et al., 2018). Tumor purity was also estimated on the basis of the cellular frequency of the dominant clone. All somatic variants were assigned to their nested clones.

### Gene expression data analysis and integration

Five sets of expression data of patient samples were selected (same as our cSCC study) and downloaded from Gene Expression Omnibus: GSE45216 (Lambert et al., 2014), GSE42677 (Mitsui et al., 2014), GSE84293 (Chitsazzadeh et al., 2016), GSE2503 (Nindl et al., 2006; Padilla et al., 2010), and GSE32628 (Hameetman et al., 2013). Differential expression analyses between different groups were performed using limma R package (Ritchie et al., 2015). The Differential expression genes were defined at adjusted *P*  ≤ 0.1.

### Data availability statement

Datasets related to this article can be found at https://www.ebi.ac.uk/ega/search/site/EGAS00001004243, deposited at the European Genome-phenome Archive, which is hosted by European Bioinformatics Institute (Cambridge, United Kingdom) and the Centre for Genomic Regulation (Barcelona, Spain) (Lappalainen et al., 2015).

## ORCIDs

Jason Thomson: https://orcid.org/0000-0002-6644-0979

Findlay Bewicke-Copley: https://orcid.org/0000-0003-1292-7965

Chinedu Anthony Anene: https://orcid.org/0000-0002-3591-3358

Abha Gulati: https://orcid.org/0000-0003-1970-6145

Karin Purdie: https://orcid.org/0000-0001-7928-0743

Gareth J. Inman: https://orcid.org/0000-0002-6264-4253

Charlotte M. Proby: https://orcid.org/0000-0002-3292-4836

Irene M. Leigh: https://orcid.org/0000-0001-8536-6439

Catherine A. Harwood: https://orcid.org/0000-0002-1375-0965

Jun Wang: https://orcid.org/0000-0003-2509-9599

## Conflict of Interest

CAH reports receiving speaker honoraria from Sanofi/Regeneron and Merck and advisory board honoraria from Roche, Leo Pharma, and Pellepharm. CAH acts as the clinical trial investigator in Novartis, Principia, Leo Pharma, Pellepharm. CAH reports receiving research funding for an investigator-led clinical trial from Meda AB. The remaining authors state no conflict of interest.
